# Financial burden faced by breastfeeding mothers caring for children diagnosed with cancer in Ghana; an exploratory qualitative study

**DOI:** 10.1186/s12905-024-02931-5

**Published:** 2024-03-14

**Authors:** Margaret Marfo, Angela Kwartemaa Acheampong, Comfort Asare

**Affiliations:** School of Nursing and Midwifery, Wisconsin International University College-Ghana, Accra, Ghana

**Keywords:** Breastfeeding, Children with cancer, Financial burden, Qualitative research

## Abstract

**Background:**

When children are diagnosed of cancer, parents face varied financial issues. Among some of the identifiable factors that cause financial challenges among breastfeeding mothers include the high cost of childhood cancer care. The high cost of childhood cancer care could impede the sustainability of access to prompt care. There is paucity of literature on the financial burdens faced by breastfeeding mothers with children diagnosed with cancer in Ghana. Therefore, this study sought to explore the financial burden faced by mothers with breastfeeding children diagnosed with cancer.

**Methods:**

The study employed qualitative exploratory descriptive design. One-on-one interviews were conducted among 13 mothers with breastfeeding children diagnosed of cancer. Permission was sought for data to be recorded, transcribed concurrently and inductive content analysis done.

**Results:**

Three main themes emerged after data analysis: High cost (sub-themes; expensive medications, laboratory investigation fees, and cost of mothers’ feeding), Public support (sub-themes; appeal for funds, national health insurance scheme) and Self-financing (loans, personal savings). Most of the breastfeeding mothers narrated that high cost of childhood cancer care generated financial distress to them. They shared that the cost involved in purchasing their children’s cancer medications, paying for laboratory investigations and feeding themselves to produce adequate breastmilk to feed their children were challenging. Some of the mothers self-financed the cost of their children’s cancer care through loans and personal savings.

**Conclusion:**

Government and other stakeholders should allocate annual budget and funds towards childhood cancer care to lessen the financial burden breastfeeding mothers caring for children with cancer experience.

## Introduction

According to the World Health Organization, about 400,000 children between the ages of 0–19 are affected by cancer globally [1]. It has been recorded that leukaemia is the commonest cancer among children accounting for 28% of all cases [2]. It is estimated that in 2023, approximately 9910 children in the United States under the age of 15 will be diagnosed with cancer [3]. Globally, 254,239 to 331,993 new cases and 86,124 to 113,581 mortalities from childhood cancer were documented in 2019 [4]. In Ghana, 1, 200 children under the age of 15 years are estimated to be diagnosed with cancer annually [1]. Korle-Bu Teaching Hospital, Komfo Anokye Teaching Hospital and the Tamale Teaching Hospital are currently the tertiary hospitals in Ghana diagnosing and treating children with cancer. Every year, the Paediatric Cancer Unit of Korle Bu Teaching Hospital treats an average of 170 new cases [5]. However, 80% of children diagnosed of cancer survive after successful treatment in high income countries [6]. Contrary to high income countries success rates, the burden of childhood cancer in low-income countries is high as a result of constraints in accessing supportive care and insurance coverage as well as shortfalls in setting up national cancer control system [7, 8].

When children are diagnosed of cancer, parents face varied financial issues [9–12]. One of the identifiable factors that promote financial toxicity among mothers is the high cost of childhood cancer care [13]. The high cost of childhood cancer care can impede the sustainability of access to prompt care [13]. Several literature across the world posit that childhood cancer medications for chemotherapy are expensive hence affordability is a challenge [14–17]. It has been found that mothers, particularly those who were on maternity leave and hence were breastfeeding when their children were diagnosed with cancer, reported impediments in their employment status because they had to spend more time with their ill-stricken children thereby affecting their incomes or cash in-flows [9, 11]. A study conducted at Denmark echoed similar findings that majority of mothers’ jobs are disrupted after their children were diagnosed of cancer [18]. Unemployment during these difficult times further hampers inability of mothers to afford their children’s expensive cancer treatment [19].

The high cost of care of children with cancer also affect breastfeeding mothers’ capacity to eat nutritious meals to produce enough breastmilk to breastfeed their sick infants. Meanwhile, evidence from a meta-analysis found that breastfeeding provides potential protective role in preventing selective childhood cancer growth in children [20]. Therefore, it was further recommended that breastfeeding of infants must be prolonged for as long as possible or maintained for at least 6 months to avoid selective childhood cancer growth or spread [20]. High cost of care and low socioeconomic status lead to treatment abandonment [21–23].

Public support in the form of insurance created by the Government of India contributes to lessening the financial burden of childhood cancer care in most parts of India [24, 25]. Findings from a quantitative study to assess the effects of socioeconomic status on children with well-differentiated thyroid cancer espoused that insurance status of a child with well-differentiated thyroid cancer was linked to higher rate of diagnosis. However, among uninsured children, the time between diagnosis and treatment was prolonged (28 days) as against those with government (19 days) or private (18 days) insurance [26]. It has been reported that in the United States, access to health insurance scheme at diagnosis is associated with childhood cancer survivorship compared to children without any form of health insurance at diagnosis [27]. A retrospective study in Kenya deduced that access to health insurance especially at diagnosis lead to event-free survival among children with cancer [8]. In the absence of state insurance coverage for paediatric cancer, parents of children with cancer resort to out-of pocket payment. This in the long run has implications on the quality of life of parents [28]. The benevolence of the local community members provide the mothers with transportation to the treatment centers [29]. Other parents have managed the cost of their children’s cancer treatment by relying on their savings and leveraging their benefits and assets for loans resulting in an increased debt [30]. There is dearth of literature on the financial burdens faced by mothers with breastfeeding children diagnosed with cancer. Therefore, this study sought to explore the financial burden faced by breastfeeding mothers with children diagnosed of cancer in Ghana and how they manage to survive under such circumstances.

## Methods

### Aim, design and setting

The study aimed at exploring the financial burden faced by mothers with breastfeeding children diagnosed of cancer in Ghana as they navigated their care journey. Qualitative exploratory descriptive design was used to conduct the study. The settings for the study were Korle-bu Teaching Hospital and Komfo Anokye Teaching Hospital in Ghana specifically the Paediatric Oncology Units (PoU). These two facilities are the two public tertiary referral hospitals in Ghana. These Units are for children receiving outpatient chemotherapy and follow-up care. The above services are in addition to the in-patient services provided to children with cancer in Ghana and from other West African countries.

### Sampling and data collection procedures

Sampling is the method through which participants are voluntarily selected into a study by the researcher (Padgett, 2016). Purposive sampling method was employed to recruit participants into the study. Purposive sampling method was used to select participants because it gave the authors an opportunity to intentionally select participants who could address the subject matter which was investigated. Individualized interviews were conducted for 13 [31] breastfeeding mothers caring for children with cancer who chose to take part in the study and met the inclusion criteria. The inclusion criteria were; breastfeeding mothers whose children were diagnosed with cancer and receiving treatment at the units. This enabled the researchers gain varied insights into the perspectives and experiences of breastfeeding mothers of children with cancer. The researchers’ sought permission from authorities of the Units after ethical approval was obtained from the Institutional Review Boards of the Hospitals. The In-charges of the wards arranged for an initial engagement with the mothers who met the inclusion criteria. The purpose of the study was explained to the participants and information sheets were read to prospective participants in the language they understood. Interested participants who met the inclusion criteria and willing to participate in the study were approached individually. Consent forms were given to participants to sign. To ensure confidentiality and privacy, participants were asked not to mention their names or names of related institutions on audio. Therefore, alpha-numeric codes were used to represent participants. The researchers also ensured that, transcripts were not matched with consent forms and they were not kept together. Interview guide was used to conduct one-on-one interviews at the participants’ own time and at a convenient place to ensure privacy. Each individualized interview lasted between 30 minutes and an hour. Responses of the participants were audio-taped with the participants’ permission in order to fully capture all their views and not miss any relevant ideas participants shared. Data collection lasted over a period of two [2] months to enable the researchers ample time to purposefully recruit breastfeeding mothers caring for children diagnosed with cancer who were a minority population. Concurrent data collection and transcription was done. The concurrent data collection and transcription improved the probing questions asked in subsequent interviews until data saturation was reached. It also allowed for member checking to clarify any ambiguous statements made by any of the participants. This generated quality data. Comprehensive field notes to capture the verbal and non-verbal cues from the participants and any unexpected event during the participants’ and researchers’ interaction were kept during data collection. Data saturation was achieved on the thirteenth participant where no new information was received. This was determined by comparing the findings from the last three transcripts (11th, 12th and 13th) with the findings of the previous 10 transcripts and it was obvious that, no new themes or sub-themes were emerging after the tenth interview. The reason for saturation was due to the fact that at that point, further data collection would not have yielded any valuable additional information.

### Data analysis

The data was analysed through inductive content analysis. Qualitative content analysis is defined as an empirical, procedurally controlled inquiry of texts in their communicative environment [31, 32]. Inductive content analysis was done by transcribing data that was collected verbatim from audio to text concurrently with data collection. The process of analysing qualitative data involved coding or categorizing the data by reading and re-reading the data to make meaning of sentences and assigning descriptive labels that allowed the researchers to identify related content across the data. Three of the authors were asked to code all the transcripts independently and this yielded similar codes. After this exercise, all authors met to discuss and agree on the final codes that formed the basis for the formulation of themes and sub-themes which ensured inter-coder reliability. Set of codes or similar codes were aggregated to generate sub-themes and then the sub-themes were also grouped for major themes to emerge. The induced major themes become the main findings of the study around which the final report was presented [33, 34].

### Trustworthiness of the study

Member checking was done by contacting participants to confirm what was said during the interview to clarify ambiguous responses. These tests confirmed the validity of findings, data, and interpretations. To confirm the study’s credibility, the researchers recruited participants who met the study’s inclusion criteria and voluntarily wanted to participate in the study. Concurrent data collection and transcription were done to ensure that both verbal and non-verbal clues were collected to ensure reality. Member checking can also take place in face-to-face interviews [35]. Interview guides were used to conduct face-to-face interviews during study. Triangulation was attained by merging field notes and transcripts to precisely represent participants’ perspectives. The researchers ensured transferability by outlining the description of the study setting, population and a thorough clarification of the process used to conduct the research from the onset to the latter so that other researchers who would like to repeat the study in a similar setting would have detailed explanation of the type of setting. Biases and prejudices were avoided by the researchers by bracketing those biases. The researchers read carefully through the transcripts before interpretation to ensure confirmability. Confirmability was also ensured through audit trail. Experts in the area of cancer research were allowed to review the research findings.

### Ethical consideration

Ethical clearance was sought from the Scientific and Technical Committee and the Institutional Review Board of the Korle-Bu Teaching Hospital (KBTH-IRB/000199/2021) and Komfo Anokye Teaching Hospital (KATH IRB/AP/047/21) to recruit participants for the study and collect data. Permission letters together with the ethical approvals were sent to the gate keepers of Oncology Units of the two Hospitals. Informed consent to participate in the study was sought by explaining the aims and details about the study to the participants in the simplest of language the participant understood for participants to make informed decisions [36, 37]. Participants were informed that participation in the study was voluntary [38] and that they were at liberty to withdraw from participating at any time without being intimidated or subjecting to withdrawal of service. Consent forms were given to participants who voluntarily chose to participate in the study. The researchers ensured that participants understood the purpose of the study before signing the consent forms. Privacy was ensured by interviewing participants in a quiet and convenient places chosen by the participant to enhance free expression of their perspectives. With their permission, the interviews were recorded. Confidentiality and anonymity were ensured by using codes as participants’ names and not storing consent forms together with the transcripts to avoid any link of the transcripts with the participants.

## Results

### Participants’ background

In all, 13 participants participated in the study. Their age ranged between 18 to 49 years old. Most of them were Christians with a few Muslims. They spoke different Ghanaian languages as well as English. A few were unemployed while majority of them had jobs. Details of participants’ background have been outlined in Table 1 below:

**Table 1:** Participants’ background

ID CODE	AGE RANGE	CHILD’S AGE	RELATIONSHIP WITH CHILD	TRIBE	LANGUAGE(S) SPOKEN	RELIGION	RESIDENCE	OCCUPATION
BM1	25–30	8MONTHS	MOTHER	AKAN	TWI	CHRISTIANITY	TEMA	TRADER
BM2	30–35	4MONTHS	MOTHER	KUSASI	TWI, KUSASI	MUSLIM	NSAWAM	TRADER
BM3	30–35	18MONTHS	MOTHER	AKAN	TWI	CHRISTIAN	DOBORO	UNEMPLOYED
BM4	29–39	21MONTHS	MOTHER	EWE	EWE, TWI	CHRISTIANITY	AFRAM PLAINS	FARMER
BM5	18–28	9 MONTHS	MOTHER	BAASELI (NORTH)	TWI, BAASELI	CHRISTIANITY	OFFINSO	TRADER
BM6	18–28	11MONTHS	MOTHER	MOSE	WANGRA, TWI, ENGLISH	MUSLIM	WENCHI	HEALTH CARE ASSISTANT
BM7	28–39	19MONTHS	MOTHER	KRUSASI	KRUSASI, TWI	MUSLIM	ASHANTI MAMPONG	FARMER
BM8	29–39	12MONTHS	MOTHER	AKAN	TWI, ENGLISH	CHRISTIAN	ABUAKWAH	TRADER
BM9	29–39	3WEEKS	MOTHER	KROBO	HAUSA, TWI, KROBO, ENGLISH	CHRISTIAN	APPIADU KOKOBEN	UNEMPLOYED
BM10	40–49	15MONTHS	MOTHER	AKAN	TWI	CHRISTIAN	ADANSI ATOBIASE	TRADER
BM11	29–39	20MONTHS	MOTHER	AKUAPIM	TWI, ENGLISH	CHRISTIAN	SEFWI YOASO	SEAMSTRESS
BM12	18–28	12MONTHS	MOTHER	BONO	TWI, ENGLISH	CHRISTIAN	TACHIMAN	UNEMPLOYED
BM13	40–49	9MONTHS	MOTHER	AKAN	TWI, ENGLISH	CHRISTIAN	ATONSO	UNEMPLOYED

### Themes and sub-themes

Three main themes and seven sub-themes emerged after data analysis. Details of themes and sub-themes have been summerised in Table 2 below:

**Table 2:** Themes and sub-themes

Themes	Sub-themes
High cost	• Expensive medications •Laboratory investigation fees • Cost of mothers’ feeding
Public support	• Appeal for funds • National Health Insurance Scheme
Self-financing	• Loans •Personal savings

Source: Transcribed data

### High cost

One of the themes that emerged after the analysis of data was high cost. Participants narrated that, as breastfeeding mothers of children with cancer, the high cost of childhood cancer treatment which made it difficult for them to afford balanced meals for themselves created financial distress for them. They shared that the cost involved in purchasing their children’s cancer medications, paying for laboratory investigations and feeding themselves well in order to produce enough breast milk was high. The sub-themes that emerged out of this theme included high cost of treatment, laboratory investigation fees and cost of mothers’ feeding.

### Expensive medications

Majority of the breastfeeding mothers narrated that the cancer medications prescribed for their children were very expensive. Meanwhile, they found it difficult to afford them as a result of their financial challenges. However, they had no option but to find the money at all cost to buy the medications for their children’s treatment.

*“It’s not easy at all because of the money issues. You have to go and buy drugs when you come back another drug is there for you to go and buy and they will say they need it urgently. So, you have to go and buy it. Anyhow that you will find money to buy, you have to do so. The drugs are expensive”.* **BM1**

*“ … when you are on your way to the hospital, you are always thinking about what you will do when you are asked to buy some drugs. The drugs are expensive. I recently was asked to buy a prescribed medication for my child. I didn’t get money to buy, so I bought the cheaper one. That was what I could afford”.* **BM3**

### Laboratory investigation fees

Some of the participants reported that, the economic demands associated with care of children with cancer was high. These demands mitigated against their ability to pay for the cost of laboratory investigations ordered by the attending physicians as part of their treatment regimen. They further mentioned that, attempts to obtain financial support proved futile.

“*I have spent everything I have. His father has been the one taking care of the rest. Even with that he couldn’t get money to cater for a prescribed lab this morning. The doctors will not be able to ascertain the condition very well if the lab investigation is not done too. There is no help”.* **BM9**

*“It has been difficult in terms of finance. Everything requires money. I have even wept today because I couldn’t get money for lab investigations this morning and all attempts to get help from people I know have proven futile”.* **BM8**

### Cost of mothers’ feeding

The breastfeeding mothers expressed that they wished to eat nutritious highly meals in order to produce adequate breastmilk to feed their ill-stricken children. However, they were unable to do so as a result of the financial constraints they face.

*“If I should get money to feed well, the breast milk would flow very well.*

*Things are difficult for me. Because of the drugs I’m supposed to buy, even if I have money, I can’t use it for food for myself to keep me lactating”.* **BM3.**

*“I am supposed to eat foods with soup as well as eat enough so as to produce more breastmilk to breastfeed my child. I sometimes cannot afford it. Other times too when she is unable to eat, I also do not feel like eating”.* **BM12.**

### Public support

Another theme that emerged after data analysis was public support. Participants disclosed that the financial burden associated with caring for children with cancer was challenging. Therefore, they were ready to reach out for public support via the media. Again, they also articulated that the National Health Insurance Scheme (NHIS) has a limitation of covering the entire cost of childhood cancer treatment. They were compelled to pay for some of the prescribed medications out-of pocket. Hence, advocated for governmental intervention to get all cost incurred covered by the NHIS.

### Appeal for funds

Participants revealed that, as a result of the financial issues associated with caring for a breastfeeding child with cancer, they were ready to appeal for support from the public through the media.

“*… for the financial issues, I even told the nurses I want to go to Adom FM (radio station) to see if I could get public support. But I was told the process is quiet long. So, I would try to get such help through Television too”.* **BM5.**

*“I would like to appeal to the press so that special care and support could be given to some of us whose children are facing such situations because the problem now is with money, and I wish we get support”.* **BM9.**

### National Health Insurance Scheme

Majority of the breastfeeding mothers stated that, the NHIS was able to cover some of the cost of medications prescribed for their children but not all. Hence, they were forced to pay for such medications that were not covered under the scheme out-of pocket irrespective of the financial constraints they faced. The mothers were therefore of the view that, to ameliorate the high cost of childhood cancer treatment, the government must ensure that the NHIS covers the cost of childhood cancer treatment.

*“NHIS covers some of the cost of medications but not all, sometimes too they will say some of the drugs are not available so you would have to go and buy … The government must improve the NHIS to completely cover childhood cancer treatment”.* **BM2**

*“I have NHIS. It (NHIS) sometimes helps to pay for some of the medications but other times too when you are asked to buy a drug, it does not cover. I am pleading with the government to make it cover most of the treatment”.* **BM11**

*“They say insurance does not cover the cost of drugs; the drugs for cancer are not covered by insurance but by some philanthropists who make donations and when you are lucky enough to get the chance then it helps you out”.* **BM13**

### Self-financing

Another theme that emerged from the study was self-financing. Some of the participants disclosed that they self-financed the cost of their children’s treatment. They indicated that they relied on loans and personal savings from their previous employment to support the cost of treatment of their children.

### Loans

Majority of the participants asserted that in order to cater for the cost of their children’s cancer treatment, they resorted to borrowing monies from friends and other benevolent individuals.

*“Loans and monies from benevolent people were what I used to cater for my child in the hospital. I will pay the loans when he recovers fully and discharged”.* **BM7**

*“I went upstairs to cry for a while before calling a good friend of mine who was willing to lend me money for my child’s medications. We have been going in for loans from people. Anytime we are to visit the hospital, we have to go borrow money”.* **BM9**

### Personal savings

Some of the breastfeeding mothers narrated that they relied on their personal savings from their previous work to support the cost of their children’s cancer treatment. As a result of that, they had gone bankrupt and are financially struggling.

*“...my child’s condition has really affected my financial life. The money from my trading activities was what I used in caring for my family, and saved some money out of it. Since my child got sick, I have lost everything including what I had saved at the bank for my child’s treatment.”* **BM10**

“*I have been through a lot financially. I have used all my personal savings on my child’s treatment. It is not good at all. Anytime we come here, they take money meanwhile I am unemployed at the moment and there is no source of income”.* **BM12**

## Discussion

### To explore the financial burden of pediatric cancer care

Breastfeeding mothers caring for children with cancer disclosed that the cost of care associated with caring for a child with cancer was high which leaves them in a state of financial distress. They narrated that the cancer medications for their children’s treatment was expensive and affordability was a challenge. The mothers further intimated that paying for the various laboratory investigations their children had to undergo as well as affording decent meals in order to produce enough breast milk were difficult. Several studies corroborates that the cost of childhood cancer care is high [25, 39, 40]. The financial constraints mothers face in the course of their children’s treatment can culminate in treatment abandonment [41]. Such treatment abandonment [42], from high cost of paediatric cancer treatment can be addressed by making childhood cancer treatment cost effective [5, 43]. Findings from a qualitative study to explore the challenges of breastfeeding sick infants and children on a paediatric ward or paediatric intensive care unit espoused that lack of food and inadequate breast pump provision made breastfeeding of a sick child more challenging [44]. The financial difficulties involved with childhood cancer care could contribute to mothers not getting enough food to eat in order to produce adequate breastmilk to breastfeed their children. The high cost of childhood cancer care that mothers encounter could be attributed to the fact that childhood cancer treatment is economically draining because it comes with prolong treatment regimen, hospitalizations and re-hospitalizations. Often mothers tend to be the primary caregivers who commit a lot of time and resources to care for the sick child and by so doing, they lose their jobs as a result of the prolonged hospitalization making affordability of the high cost of care challenging. It is therefore recommended that government and other stakeholders allocate annual budget and funds towards childhood cancer care. This will alleviate the financial burdens mothers face and promote global access to childhood cancer treatment.

### To explore how mothers manage under such circumstances

The breastfeeding mothers of children with cancer advocated for public support through the National Health Insurance Scheme (NHIS) to cover the entire cost of childhood cancer treatment. They believed that the insurance coverage for their children will reduce the financial burden associated with caring for their children with cancer. They were ready to appeal for public support through the media to aid in the care of their children with cancer. The impact of public support for childhood cancer care through NHIS coverage has been extensively reported in previous studies [45–49]. This prevents out-of-pocket payment [9, 28] that leads to impoverishment and other financial burden. Insurance coverage for childhood cancer care result in improved health outcomes as well as contributing to high survival rate among children with cancer. Conversely, it was stipulated in a study conducted in USA that regardless of insurance, greater risk of deaths were reported among black adolescents and young adults with cancer compared to whites [50]. Other studies have illuminated that some parents of children with cancer rely on the support and benevolence of the local community who provide them with money or transportation to the treatment centers [29]. Mothers caring for breastfeeding children with cancer’s request for public support to care for their sick children may be due to the fact that, as breastfeeding mothers with sick children, they need to stay with their sick children at the hospital to nourish them frequently with enough breastmilk to boost the immunity of the child. Empirical studies have reported on the therapeutic effect of breastmilk on childhood cancer and child’s immunity [51–53]. Mothers who understand the benefit of breastfeeding are unable to leave their breastfeeding sick children in an attempt to attend to their economic activities to raise some funds to support the care of their children. Hence NHIS coverage of childhood cancer treatment and other cost by the government and support from the general public and the community would go a long way to ameliorate the financial burdens such vulnerable women go through in the course of caring for their children.

Majority of the mothers in this current study reported that, they self-financed the cost of their children’s treatment. They disclosed that, they borrowed money from friends in the form of loan to take care of their children’s treatment needs and cater for other hidden expenses [54]. Borrowing money from family and friends, selling property or mortgaging homes, taking on loans or credit card debt and reduced spending have been reported as additional coping strategies adapted by families of children with cancer [55]. Some also relied on their personal savings from previous employment incomes to support the cost of treatment of their children. Literature has documented that some parents utilize their personal savings and leverage their benefits and assets for loans to support the cost of their children’s cancer treatment [30]. When mothers continue to rely on their savings and loans, they experience financial challenges. It was more obvious among mothers whose employment terminated yet had to spend all their savings on their children’s treatment which led to a great deal of financial distress [9]. Breastfeeding mothers struggled to find their own financial resources for their children’s cancer treatment because of limited allocation of funds for childhood cancer in Ghana. Again, mothers’ maternal instincts and the love for their children propels them to go all out to raise funds and spend their last penny to save their lives. Therefore, in addition to advocating for governmental support, philanthropists, corporate and faith-based organizations should offer aids to parents typically breastfeeding mothers caring for children with cancer where possible.

## Conclusion

It came to light in this current study that, mothers of breastfeeding children diagnosed with cancer found the cost of childhood care high. They articulated that, childhood cancer medications were extremely expensive. In addition, they stated that paying for the series of laboratory investigations the children go through was difficult. Affordability of decent meals in order to produce enough breastmilk to breastfeed their sick children was a constraint for them as well. The mothers highlighted that public support in the form of complete national health insurance coverage for childhood cancer care will alleviate their financial burdens. They were ready to appeal for public funds via the mass media to support the cost of their children’s cancer treatment. Most of the mothers self-financed the treatment of their children through loans and personal savings. Findings from this study asserts the need for the Government of Ghana to urgently address the financial burden associated with caring for infants with cancer. Therefore, government and other stakeholders must allocate annual budget and funds towards childhood cancer care to lessen the financial burden breastfeeding mothers caring for children with cancer experience.

### Potential impact of the findings

The authors envisage that illuminating these financial challenges mothers face will bring the attention of stakeholders to commit financial resources towards paediatric cancer care to ease the financial distress mothers go through. In addition to this, the findings of this study may inform policies on treatment regimen regarding paediatric oncology. Other researchers may conduct further studies on issues such as; crowd funding for paediatric oncology and counselling tools to support breastfeeding mothers caring for children with cancer.

### Recommendations

Based on the findings of this study, the following recommendations are made:

- The Ghana National Health Insurance Scheme should cover all paediatric cancer cases.
- Special provision should be made to feed breastfeeding mothers together with their children who are admitted at child oncology centers.
- Specialized counselling centers should be attached to all paediatric oncology centers to assist mothers cope with the stress of caring for children with cancer.
- Hospitals should provide restrooms that can accommodate breastfeeding mothers at various oncology departments

### Strengths and limitations

The strength of this research was that, it brought in-depth perspectives of the financial burden of paediatric oncology care to bare. The inter-coder reliability which was ensured strengthened the findings of the research. On the other hand, this study was limited to only breastfeeding mothers caring for children with cancer. Further studies can concentrate on other care givers of children with cancer admitted to paediatric oncology departments across Ghana.

## Data Availability

The datasets for the analysis of this study can be attained from the corresponding author on reasonable request.
